# Cardiac FGF23: a new player in myocardial infarction

**DOI:** 10.15190/d.2019.10

**Published:** 2019-09-30

**Authors:** David Schumacher, Alexander Schuh

**Affiliations:** Department of Anesthesiology, University Hospital, RWTH Aachen, Germany; Department of Cardiology and Pulmonology, Medical Faculty, RWTH Aachen University, Germany

**Keywords:** Fibroblast Growth Factor 23, FGF-23, myocardial infarction, inflammation, cytokines, heart failure, ischemic heart disease, fibrosis, cardiac hypertrophy.

## Abstract

Fibroblast Growth Factor 23 (FGF23) is a hormone involved in phosphate metabolism. It is known that FGF23 is increased in different pathologies including chronic kidney disease, heart failure or X-linked hypophosphatemia and directly correlates with negative outcome and mortality in severe diseases. However, the role of FGF23 in cardiovascular pathologies is still under debate. This review summarizes the current knowledge about the role of FGF23 in ischemic heart diseases, such as myocardial infarction.

## **1. Introduction**

Despite a significant progress in diagnostics and therapeutic strategies, ischemic heart disease is still the leading cause of death worldwide according to the World Health Association^1^. Myocardial infarction is the most feared complication of ischemic heart disease. It occurs when blood flow to the cardiac muscle decreases or is stopped^2^. The occlusion of a coronary artery is mostly caused by the rupture of an atherosclerotic plaque^2^. The following mismatch between oxygen demand and supply leads to the death of cardiomyocytes^2^. After myocardial infarction complex healing processes lead to the formation of a scar tissue, and eventually to heart failure^3-5^. The inflammatory phase with abundant infiltration of neutrophils and macrophages is followed by a proliferative phase with formation of extracellular matrix by myofibroblasts and neoangiogenesis^3-5^. Imbalance in the healing processes can lead to fibrosis, heart failure or ventricular rupture^3-5^. There are many factors involved in the regulation of these processes. Interestingly, Fibroblast Growth Factor 23 (FGF23) was shown by several studies to be a positive predictor for mortality or cardiovascular complications in heart failure, chronic kidney disease and sepsis^6-10^.

Fibroblast Growth Factor 23 (FGF23) is a hormone mainly produced in osteocytes. It increases renal phosphate excretion^11^. Further it decreases calcitriol and parathormone synthesis^11^. The main stimulators of FGF23 synthesis are phosphorus load and active vitamin D^12,13^. Additionally, other factors such as calcium, iron, parathormone and inflammation influence the FGF23 synthesis^14^. FGF23 is increased in different pathologies including chronic kidney disease, heart failure or X-linked hypophosphatemia^6,15,16^. Actually, many studies show a positive correlation between high amounts of FGF23 in serum and negative outcome and mortality in severe diseases^6,8,17,18^. FGF23 seems to be a positive predictor for mortality or cardiovascular complications in chronic kidney disease, heart failure and sepsis^6,7,9,10^. 

During chronic kidney disease the amount of FGF23 in serum is increased up to 1000 fold compared to normal^15^. It is due to the high phosphorus load and has the goal to increase the phosphate excretion^19^. Further this leads to vitamin D and calcium deficiency^20^ and consequent to renal osteodystrophy. During chronic kidney disease, FGF23 is upregulated in osteocytes and released into blood. Several studies showed that increased serum FGF23 during chronic kidney disease is a reliable prognostic marker^9,18^. FGF23 positively correlates with outcome and cardiovascular mortality^9^.

FGF23 is less studied in cardiovascular pathologies. It is known from experimental studies that chronically increased FGF23 is able to induce pathological left ventricular hypertrophy^21^. FGF23 increases the calcium influx and contractility of cardiomyocytes in vitro, leading to cardiomyocyte hypertrophy^22,23^. New studies showed that FGF23 is increased during heart failure and correlates with cardiac complications and mortality^6,10^. Actually, different clinical studies are investigating if FGF23 is a reliable prognostic marker in heart failure (**Table 1** shows a summary of the ongoing studies). Thus, this review aims to summarize the current knowledge about FGF23 in myocardial infarction.

**Table 1:** Clinical studies investigating FGF23 in heart failure *https://clinicaltrials.gov*

Study Title	Location	Status	Primary Outcome
Intravenous Iron in patients With Heart failure and Reduced Ejection fraction (HFREF) plus Iron deficiency	Department of Medicine, Division of Cardiology, Pulmonary Diseases and Vascular Medicine at the University Hospital, RWTH Aachen, Aachen, NRW, Germany	completed	Change of FGF23 in blood after infusion of 1000 mg ferric carboxymaltose
Iron Deficiency and FGF23 Regulation in chronic kidney disease and heart failure	Northwestern University, Chicago, Illinois, United States	completed	Change of FGF23 in plasma after Iron Sucrose therapy
New Heart Failure Biomarkers in Early Stage Chronic Kidney Disease-Mineral and Bone Disorder	Research Laboratory (LR12SP18) University of Monastir Tunisia, Tunisia and Research Unit (UR17ES29) Faculty of Pharmacy, Monastir	completed	Difference of FGF23 in blood in patients with heart failure versus patients without heart failure
Cardiorenal Risk Stratification Pilot Study (CRiSPS): Using FGF-23 as a Risk Stratification Biomarker in Patients with Heart Failure and Chronic Kidney Disease as a Predictor of 1-year Morbidity and Mortality Risk	Coney Island Hospital, Brooklyn, New York, United States	recruiting	Mortality, worsening renal or cardiac function End-Stage Renal Disease Progression in patients with heart failure with or without chronic kidney disease
Time Course of Circulating Myocardial Biomarkers After a TASH Procedure	Aachen University Hospital; Medical Clinic I - Cardiology, Pneumology, Angiology and Internal Intensive Medicine, Aachen, NRW, Germany	recruiting	Time course of FGF23 in blood in patients with hypertrophic obstructive cardiomyopathy (HOCM) before and after Transcoronary Ablation of Septal Hypertrophy (TASH)
New Biomarkers in Heart- and Renal Failure: Cohort Study for Assessing Prognosis in Acute Coronary Syndrome and Acute/Chronic Cardiovascular and Renal Failure by Means of Fibroblast Growth Factor 23	Aachen University Hospital; Medical Clinic I - Cardiology, Pneumology, Angiology and Internal Intensive Medicine, Aachen, NRW, Germany	recruiting	Survival after recording on the intermediate care station following myocardial infarction

## **2. FGF23 in myocardial infarction**

**Figure 1:**
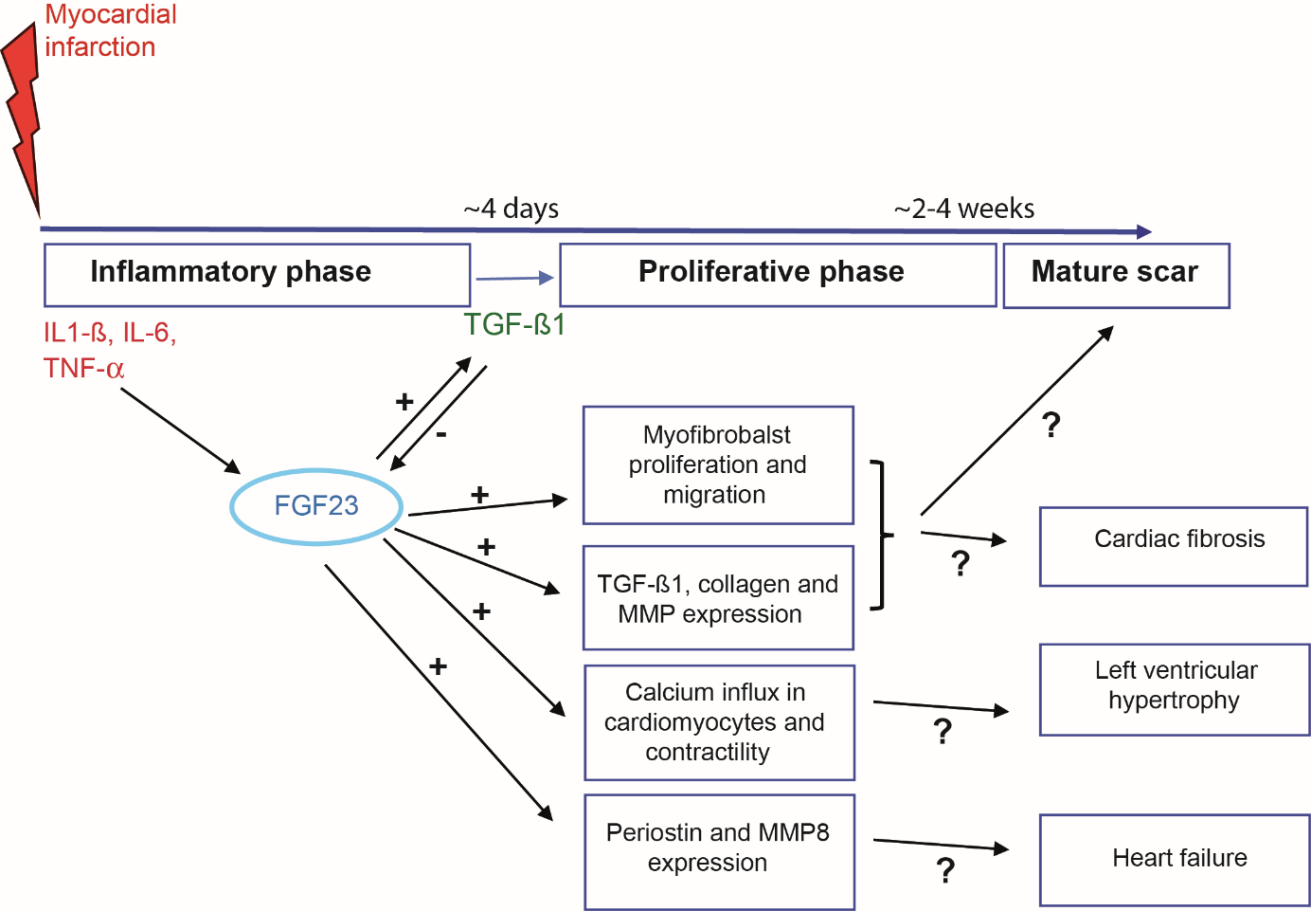
Overview of the FGF23 model of action in myocardial infarction During the inflammatory phase following myocardial infarction, IL-1ß, IL-6 and TNF-α increase the expression of FGF23. FGF23 leads to increased migration and proliferation of myofibroblasts and increases the expression of TGF-ß1, collagen and MMPs, but also expression of periostin and MMP8. Interestingly, periostin and MMP8 seem to be markers for heart failure and cardiac remodelling. While FGF23 induces the production of TGF-ß1, the increase of TGF-ß1 at the end of the inflammatory phase decreases the FGF23 expression, probably in a negative feedback mechanism. Longer or chronic increases of FGF23 after myocardial infarction could lead to fibrosis, left ventricular hypertrophy through increase of calcium influx and increased contractility of cardiomyocytes.

The role of FGF23 in myocardial infarction is not clear. While it is believed that FGF23 source are osteocytes^11^, our new study showed that FGF23 is also produced in cardiac fibroblasts following myocardial infarction^24^. It seems that cardiac fibroblasts produce FGF23 during the inflammatory phase through stimulation with Il-6 (Interleukin 6), Il-1ß (Interleukin 1ß) and TNF-α (Tumor Necrosis Factor α) , whereas TGF-ß (Transforming Growth Factor ß) inhibits the expression of FGF23 later during the proliferative phase^24^. This suggests that FGF23 could potentially play a major role in healing after myocardial infarction. Indeed, our group and others could identify potential roles of FGF23 during myocardial infarction. On one hand FGF23 increases calcium influx in cardiomyocytes^23^, which leads to increased myocardial contractility and hypertrophy^22,23^. In that way, local FGF23 possibly helps to transiently compensate the loss of contractile tissue from the infarcted area through increased contractility of the remote area after myocardial infarction. On the other hand, FGF23 seems to increase migration and proliferation of fibroblasts^24,25^, which are responsible for preserving the mechanical tissue integrity and scar formation. FGF23 increases the expression of profibrotic genes such as collagen or TGF-ß ^24,25^. **Figure 1** gives an overview of the FGF23 model of action in myocardial infarction. Clear clinical evidence concerning the role of FGF23 during myocardial infarction is lacking. Still, a small study showed an increase of FGF23 in serum after myocardial infarction^26^.

Furthermore, FGF23 significantly up-regulates factors that have been shown to be heart failure biomarkers or marker for cardiac remodelling, such as periostin and MMP8 (Matrix Metalloproteinase 8)^24,27-29^. However, the meaning of these findings remains unclear.

FGFR1c (Fibroblast Growth Factor Receptor 1c) and FGFR4 (Fibroblast Growth Factor Receptor 4) are the most abundant receptors for FGF23 in heart^24,30^. FGFR4 is well-known to mediate the hypertrophic effects of FGF23 on cardiomyocyte^30^, whereas FGFR1c probably mediates the profibrotic effects on myofibroblasts and macrophages^24^. However, the exact mechanisms remain to be elucidated.

## **3. Conclusion and perspective**

These new data showing a cardiac expression of FGF23 during myocardial infarction opens new fields of investigation. First of all, more studies are needed to clarify the exact role of FGF23 during myocardial infarction *in vivo*. It is crucial to determine whether or not FGF23 is a potential target to improve cardiac function and healing after myocardial infarction. Whereas chronically high FGF23 in chronic kidney disease is detrimental, acute elevation of FGF23 in myocardial infarction might be beneficial. Since FGF23 knockout mice are not suitable for myocardial infarction experiments due to the increased weakness and sick phenotype, other experiments are needed. Conditional gene knockouts or antibody therapies could be a possibility to elucidate the exact role of FGF23. Moreover, FGF23 in serum might be a reliable marker to predict the outcome after myocardial infarction. Finally, we should investigate the role of cardiac FGF23 in other cardiac diseases such as heart failure. In conclusion, cardiac FGF23 represents a promising new field of research.

## **KEY POINTS**

**◊** **Cardiac FGF23 expression increases directly after myocardial infarction, potentially promoting the (1) proliferation and migration of cardiac myofibroblasts, and (2) calcium influx, contractility and hypertrophy of cardiomyocytes**

**◊** **Effects of FGF23 are mediated through FGFR4 (hypertrophy) and FGFR1c (fibrosis)**

**◊** **TGF-ß decreases the expression of cardiac FGF23**
